# Pepsin promotes IL-8 signaling-induced epithelial–mesenchymal transition in laryngeal carcinoma

**DOI:** 10.1186/s12935-019-0772-7

**Published:** 2019-03-20

**Authors:** Jia-Jie Tan, Lu Wang, Ting-Ting Mo, Jie Wang, Mei-Gui Wang, Xiang-Ping Li

**Affiliations:** 1 Department of Otolaryngology, Head and Neck Surgery, Nanfang Hospital, Southern Medical University, 1838 Guangzhou Avenue North, Guangzhou, 510515 China; 2 Department of Otolaryngology, Gaoyao District Traditional Chinese Medicine Hospital of Zhaoqing, No.3 of FuQian Avenue, Zhaoqing, 526100 Guangdong China

**Keywords:** Laryngopharyngeal reflux, Laryngeal carcinoma, Pepsin

## Abstract

**Background:**

Laryngopharyngeal reflux (LPR), with its increasing morbidity, is attracting considerable attention. In recent years, the causal role between LPR and laryngeal carcinoma has been debated. The main harmful component of LPR is pepsin, which has been shown to induce mucosal inflammation by damaging the mucous membrane. Thus, pepsin is linked to an increased risk of laryngeal carcinoma, although the potential mechanism remains largely unknown.

**Methods:**

The human laryngeal carcinoma cell lines Hep-2 and Tu212 were exposed to different pepsin concentrations and the morphology, proliferation, migration, secretion of inflammatory cytokines, and epithelial–mesenchymal transition (EMT) of the cells were assessed. To evaluate whether interleukin-8 (IL-8) had a causal relationship with pepsin and EMT, an IL-8 inhibitor was used to suppress IL-8 secretion during pepsin exposure and the expression of EMT markers, cell proliferation, and migration were analyzed.

**Results:**

Pepsin promoted proliferation, colony formation, migration, and IL-8 secretion of Hep-2 and Tu212 cells in vitro. Furthermore, increased pepsin concentrations changed the morphology of Hep-2 and Tu212 cells; levels of the epithelial marker E-cadherin were reduced and those of mesenchymal markers vimentin and β-catenin and the transcription factors snail and slug were elevated. A similar effect was observed in laryngeal carcinoma tissues using immunohistochemistry. IL-8 level was reduced and EMT was restored when pepsin was inhibited by pepstatin. EMT was weakened after exposure to the IL-8 inhibitor, with significant reduction in pepsin-induced cell proliferation and migration.

**Conclusions:**

Pepsin may induce EMT in laryngeal carcinoma through the IL-8 signaling pathway, which indicates that it has potential role in enhancing cell proliferation and metastasis of laryngeal carcinoma.

**Electronic supplementary material:**

The online version of this article (10.1186/s12935-019-0772-7) contains supplementary material, which is available to authorized users.

## Background

Laryngopharyngeal reflux (LPR) is the retrograde flow of gastric contents into the upper aerodigestive tract, which causes a variety of signs and symptoms in the throat [1]. Extensive research has shown that LPR is closely related to chronic throat inflammation [2], development of vocal cord polyps [3], and other benign diseases. Recently, LPR has attracted increasing attention as a risk factor for laryngeal cancer.

As it is a common malignant otorhinolaryngologic tumor, laryngeal carcinoma is considered to be closely related to smoking and drinking [4]. However, in Western countries, controlling behaviors associated with these risk factors has not significantly lowered the incidence of laryngeal carcinoma, suggesting the existence of other causes [5, 6]. Certain recent studies have supported the hypothesis that LPR is an independent risk factor in the development of laryngeal cancer [7, 8]. However, other studies support the opposite view [9, 10]. Whether LPR plays a key role in laryngeal cancer development is still controversial, partially because LPR diagnosis currently lacks a unified standard [11–13], which renders demonstration of the correlations between reflux and laryngeal cancer difficult.

In gastroesophageal reflux disease, acid damages the esophageal epithelium. In LPR, reflux is dominated by weak acidic reflux in both upright and supine positions [14]; however, nonacid refluxes, such as those of pepsin and bile acid, require further consideration. Pepsin, the main harmful component of LPR, normally exists only in the stomach, but numerous recent studies have reported it in the trachea, lung tissue, nasal mucosa, middle ear secretions, and saliva of a reflux patient [15–18]. It is widely accepted that pepsin can act as a molecular marker of reflux [19, 20]. However, only few studies have shown that pepsin in LPR contributes to the development of laryngopharyngeal carcinogenesis [21, 22], although the relevant molecular mechanism is largely unknown.

Recent studies [23, 24] have shown reflux to be associated with epithelial–mesenchymal transition (EMT). However, the contributions of LPR to laryngeal carcinoma carcinogenesis via EMT have not yet been characterized. Considering that LPR includes pepsin-containing fluids, we recently observed that pepsin expression in laryngeal tissue increases in patients with laryngeal carcinoma [25]. In the present study, we attempted to determine whether pepsin induced laryngeal carcinoma via EMT and whether it facilitated the malignant development of laryngeal cancer.

## Materials and methods

### Tissue specimens and cell culture

This study was performed in accordance with institutional ethical guidelines and was approved by the Ethics Committee of the Nanfang Hospital. Informed written consent was obtained from each patient. Specimens from 87 patients with laryngeal carcinoma (two women and 85 men, aged 40 to 86 years) were collected from the laryngeal carcinoma tissue bank of the Nanfang Hospital. The tissue specimens were routinely frozen in liquid nitrogen, fixed in 4% paraformaldehyde, embedded in paraffin, and sectioned according to routine procedures.

The American Type Culture Collection (ATCC) human laryngeal carcinoma cell line Tu212 was purchased from Guangzhou Juyan Biological Technology (Guangzhou, China) and Hep-2 was purchased from Shanghai Aolu Biological Technology (Shanghai, China). The cells were cultured in Roswell Park Memorial Institute (RPMI)-1640 medium (Gibco, USA) supplemented with 10% fetal bovine serum (FBS; Gibco) and 1% penicillin/streptomycin. The cells were maintained at 37 °C in a humidified 5% CO_2_ atmosphere. Porcine pepsin (Sigma-Aldrich, St Louis, MO, USA) was used for pepsin exposure. The pepsin inhibitor pepstatin A and the interleukin-8 (IL-8) inhibitor SB225002 were synthetized by Selleckchem (Shanghai, China).

### 5-ethynyl-2′-deoxyuridine (EdU) proliferation assay

Hep-2 and Tu212 cells were seeded in 96-well plates (4 × 10^4^/well) and exposed to different concentrations of pepsin (0 mg/ml, 0.1 mg/ml, or 1 mg/ml) at pH 7 for 2 h. The working concentration of pepsin (0.1 mg/ml and 1 mg/ml) has been used previously [21, 22, 26–28]. After exposure to pepsin, the cells were cultured in complete RPMI-1640 medium for 24 h and cell proliferation was detected using an EdU kit (RiboBio, Guangzhou, China) according to the manufacturer’s instructions. Images were analyzed using digital microscopy and the cells were counted using Image J software.

### Colony-forming assays

Hep-2 and Tu212 cells were seeded in 6-well plates and exposed to 0 mg/ml, 0.1 mg/ml, or 1 mg/ml pepsin at pH 7 twice a week for 2 h for 18 days. The cells were maintained in complete RPMI-1640 medium when not being treated with pepsin. Colonies containing > 50 cells were fixed with 4% paraformaldehyde and stained with 0.5% crystal violet.

### Cell cycle experiment

Hep-2 and Tu212 cells were inoculated in 6-cm dishes. The cultures were divided into three groups and treated with culture media containing different concentrations of pepsin as described above. Cell-cycle distribution was analyzed using propidium iodide staining (Keygentec, Nanjing, China) and flow cytometry (Becton–Dickinson, San Jose, CA, USA).

### Transwell migration assay

Hep-2 and Tu212 cells suspended in 100 µl serum-free medium were seeded into the upper chambers of each insert (24-well insert; pore size, 8 μm; Corning, Corning, NY, USA) and 600 μl medium containing 10% fetal bovine serum (FBS) was added to the lower chamber. After 12 h, the medium in the lower chamber was replaced with 10% FBS and different concentrations of pepsin and incubated for 2 h. After 24 h, the cells under the bottom membrane were stained with crystal violet. The migrating cells in five randomly selected fields at a magnification of 200× were imaged using digital microscopy and counted.

### Cytometric bead array (CBA)

Hep-2 and Tu212 cells were seeded into 6-well plates and exposed to different concentrations of pepsin at pH 7 for 2 h. The concentrations of cytokines IL-8, IL-6, IL-10, IL-1β, IL-12p70, and tumor necrosis factor (TNF) in the serum were measured using a Becton–Dickinson CBA software following a standard protocol. The data from the standards and experimental samples were analyzed using an LSR Fortessa flow cytometer (Becton–Dickinson) and FCAP Array software.

### Extraction of total RNA and quantitative reverse transcription PCR (qRT-PCR)

Hep-2 and Tu212 cells were inoculated into 6-cm dishes and exposed to different concentrations of pepsin in the culture medium. The cells were continuously stimulated for 2 h each day for 5 days. Total RNA was extracted from the cells using RNAiso Plus (Takara, Shiga, Japan) and reverse transcribed into complementary DNA (cDNA) using an All-in-One first-strand cDNA synthesis kit (GeneCopoeia Inc., USA). The SYBR green method (GeneCopoeia Inc) was used for PCR amplification. Relative quantification using the 2^−∆∆Ct^ method was used to determine the mRNA expression levels of EMT epithelial marker E-cadherin and mesenchymal markers vimentin and β-catenin. The housekeeping gene β-actin was used as an internal control. The primers included GAPDH forward (5′-AAGAAGGTGGTGAAGCAGGC-3′) and reverse (5′-TCCACCACCCAGTTGCTGTA-3′), E-cadherin forward (5′-CCCGGGACAACGTTTATTAC-3′) and reverse (5′-GCTGGCTCAAGTCAAAGTCC-3′), vimentin forward (5′-TGCTTCAGAGAGAGGAAGCCGAA-3′) and reverse (5′-ACGTGCCAGAGACGCATTGTCA-3′), and β-catenin forward (5′-GACCAGCTCTCTCTTCAGAACAGA-3′) and reverse (5′-GTTCTCCCTGGGCACCAATA-3′).

### Western blot analysis

Hep-2 and Tu212 cells were inoculated into 6-cm dishes and exposed to different concentrations of pepsin in the culture medium. The cells were stimulated continuously for 2 h each day for 5 days. Total protein was extracted from the cells and the samples were separated using 10% sodium dodecyl sulfate-polyacrylamide gel electrophoresis (SDS-PAGE) and then electrophoretically transferred to polyvinylidene difluoride (PVDF) membranes (Millipore). The membranes were incubated overnight with rabbit monoclonal antibodies against human E-cadherin (1:1000; Cell Signaling Technology, USA), vimentin (1:100; Cell Signaling Technology), β-catenin (1:100; Cell Signaling Technology), snail (Cell Signaling Technology), and slug (Cell Signaling Technology) transcription factors and detected using chemiluminescence. An antibody specific for β-actin (1:10,000; Kangcheng Inc, Shanghai, China) was used as an internal control.

### Immunofluorescence assay

Hep-2 and Tu212 cells were inoculated in 6-well dishes and exposed to different concentrations of pepsin in culture medium. The cells were continuously stimulated for 2 h each day for 5 days, fixed with 4% paraformaldehyde for 20 min, permeabilized with 0.2% Triton X-100 for 10 min, blocked for 1 h with goat serum, and incubated at 4 °C overnight with primary antibodies against E-cadherin and vimentin. The nuclei of the cells in the confocal dishes were counterstained with 4′,6-diamidino-2-phenylindole (DAPI) and imaged using an inverted microscope.

### Immunohistochemistry

Paraffin-embedded tumor specimens from patients with laryngeal carcinoma were sectioned (4 μm) and incubated with primary antibodies against pepsin, E-cadherin, vimentin, β-catenin, and IL-8 (Cell Abcam, USA). Staining was repeated at least twice in sequential sections from the same tissue blocks and the stained sections were reviewed and classified by two pathologists. The proportion of positive cells per specimen was evaluated quantitatively and scored as follows [29]: ≤ 1% stained cells, 0; 2–25%, 1; 26–50%, 2; 51–75%, 3; and > 75%, 4. Staining intensity was scored as follows: no staining, 0; weak, 1; moderate, 2; and strong, 3. The total score (0–12) was calculated by multiplying the score of stained cells by the score of staining intensity, and was graded as negative (−; score: 0–1), weak (+; 2–4), moderate (++; 5–8), and strong (+++; 9–12).

### Statistical analysis

The SPSS 19.0 software was used for statistical analysis. Analysis of variance was performed to compare data from the EdU assays, clone formation experiments, cell cycle experiments, cell migration assays, CBA assays, and qPCR analysis. Wound scratch assays were analyzed with a factorial design analysis of variance. Expression levels of pepsin, IL-8, E-cadherin, vimentin, and β-catenin in the laryngeal carcinoma specimens as determined by immunohistochemistry were compared using Spearman correlation analysis. *P*-values < 0.05 were considered statistically significant.

## Results

### Pepsin promoted proliferation and the migratory capacity of laryngeal squamous cell carcinoma cells in vitro

**Fig. 1:**
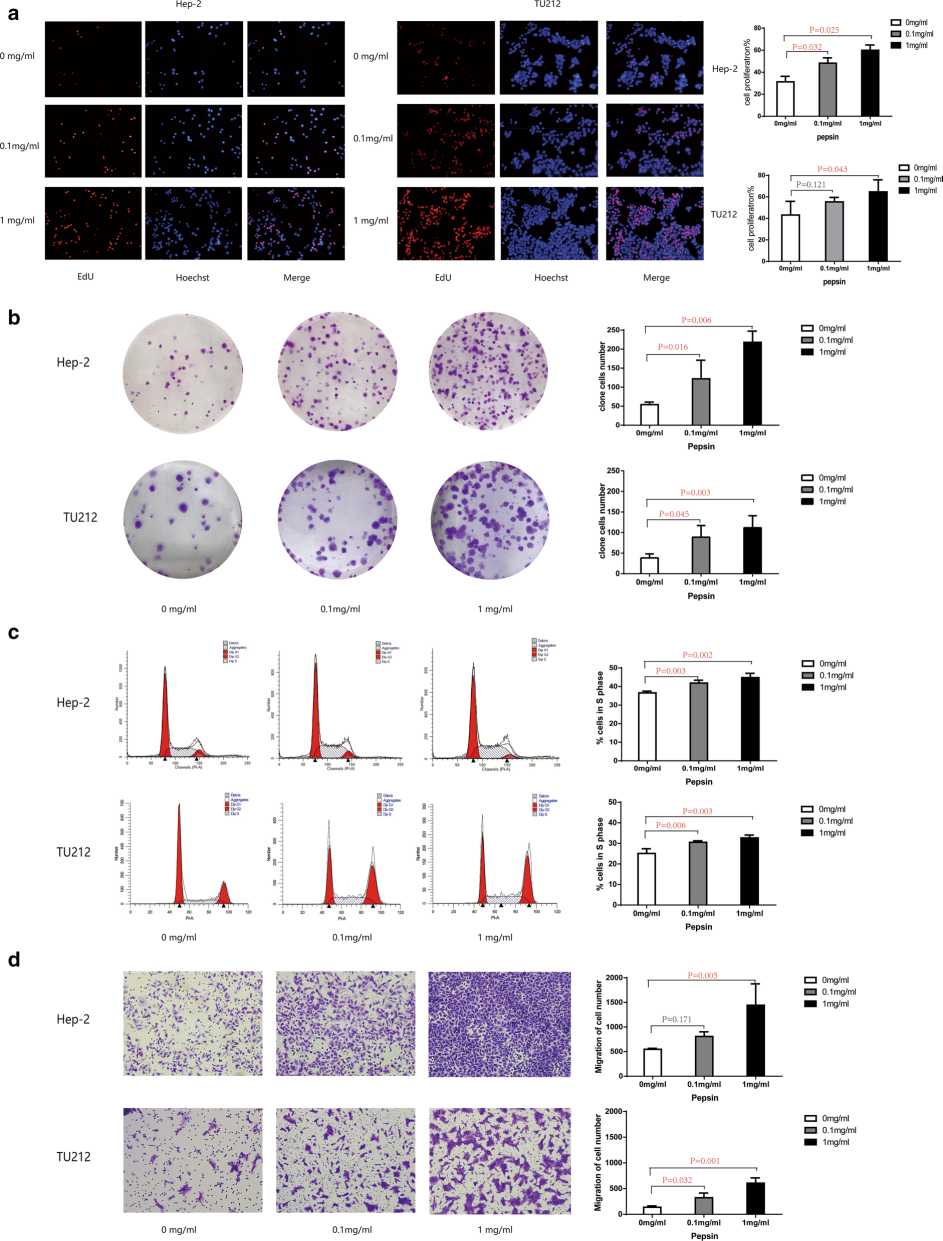
Proliferative ability of Hep-2 and Tu212 cells treated with different concentrations of pepsin. **a** Effect of different concentrations of pepsin on proliferation of Hep-2 and Tu212 cells measured using Edu assays (magnification, ×200). **b** Representative images of the colony formation assay of Hep-2 and Tu212 cells at different pepsin concentrations. **c** Representative histograms of cell cycle assays showing the percentage of Hep-2 and Tu212 cells in S phase at different pepsin concentrations. **d** Migratory properties of Hep-2 and Tu212 cells exposed to different concentrations of pepsin were analyzed using transwell migration assays (magnification, ×200). *P < 0.05 compared to that of the controls

The EdU assays showed that cell proliferation increased by 1.55- and 1.92-fold, and by 1.28- and 1.49-fold when the Hep-2 and Tu212 cells were treated with 0.1 mg/ml and 1 mg/ml pepsin, respectively, compared to that of cells not treated with pepsin, (*P* = 0.025 and *P* = 0.043; Fig. 1a). Similar results indicated that 0.1 mg/ml and 1 mg/ml pepsin increased the colony formation ability by 2.3- and 4.3-fold, and by 2.3- and 2.9-fold, respectively, compared to that of cells not treated with pepsin (*P* = 0.006 and *P* = 0.003; Fig. 1b). Furthermore, cell cycle distribution showed that the percentage of cells in the S-phase increased concurrently with increasing pepsin concentrations (*P* = 0.002 and *P* = 0.003; Fig. 1c).

Next, we investigated the cellular migration-inducing ability of pepsin. Transwell migration arrays revealed dramatic increases in cell motility, with 1.47- and 2.63-fold, and 2.35- and 4.40-fold increase for cells treated with 0.1 mg/ml and 1 mg/ml pepsin, respectively, compared to that of untreated Hep-2 and Tu212 cells (*P* < 0.001; Fig. 1d).

### Pepsin altered cytokine production by laryngeal squamous cell carcinoma cells in vitro and in laryngeal carcinoma tissues

**Fig. 2:**
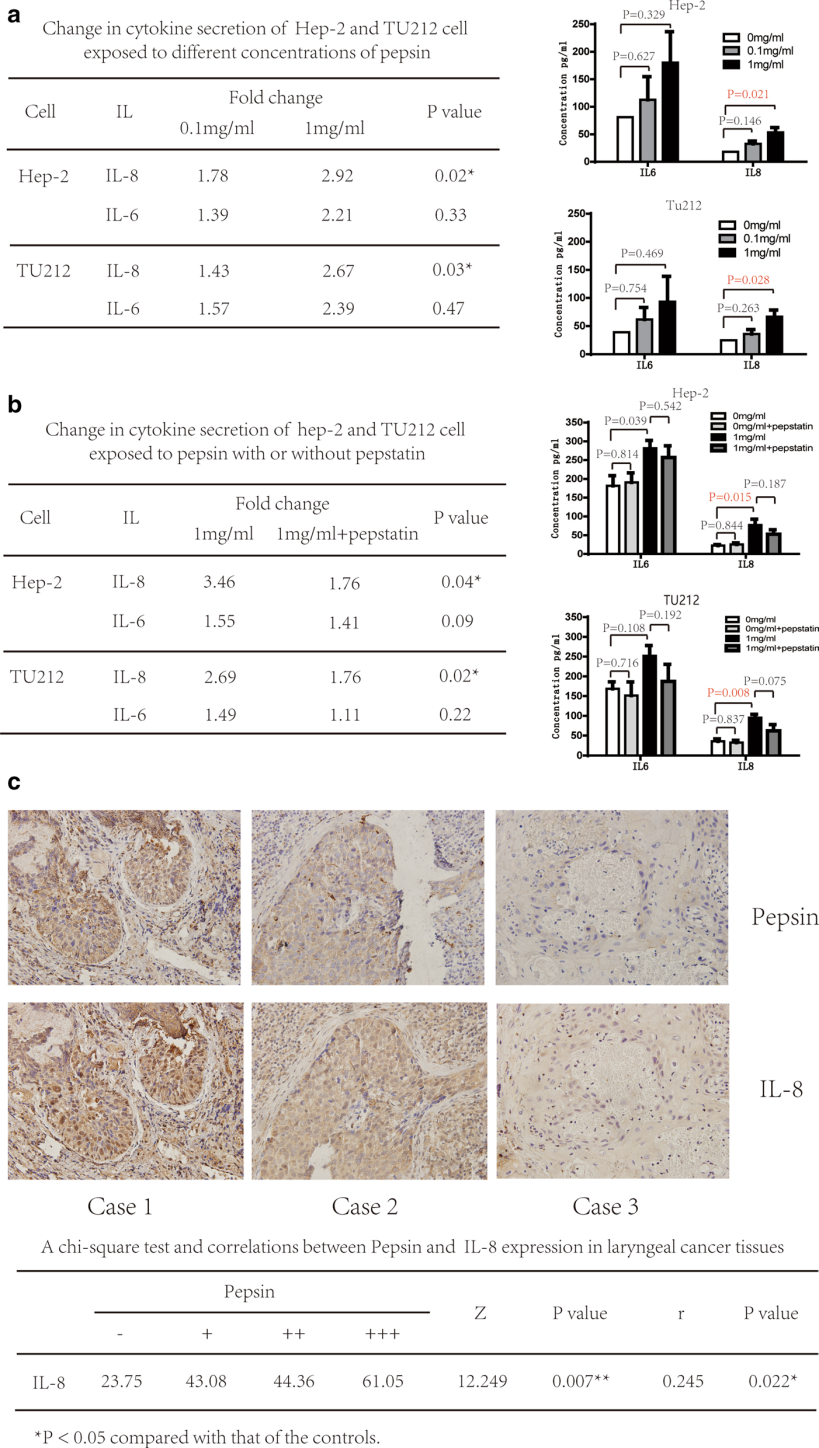
Expression of IL-8 after pepsin exposure. **a** Change in inflammatory cytokine expression of Hep-2 and Tu212 cells exposed to different concentrations of pepsin measured using the CBA assay. Production of IL-8 and IL-6 by Hep-2 and Tu212 cells at different pepsin concentrations. **b** Change in inflammatory cytokine expression of Hep-2 and Tu212 cells exposed to pepsin with/without pepstatin measured using CBA assays. Production of IL-8 and IL-6 by Hep-2 and Tu212 cells at different concentrations of pepsin. **c** Photomicrographs representative of the immunohistochemical analyses of pepsin and IL-8 in tissue specimens from three patients with laryngeal carcinoma (magnification, ×400). *P < 0.05 compared to that of the controls

To determine whether pepsin was able to alter the expression of inflammatory cytokines, which may contribute to epithelial damage, we investigated cytokine production following stimulation with pepsin. Results demonstrated that IL-8 levels increased by 1.78- and 2.92-fold, and by 1.43- and 2.67-fold with 0.1 mg/ml and 1 mg/ml pepsin, respectively (*P* = 0.02 and *P* = 0.02). There was also a trend toward elevation of IL-6 levels by pepsin, although the observed differences did not reach statistical significance (Fig. 2a). Levels of IL-10, IL-1β, IL-12p70, and TNF showed no significant difference between cells treated or not treated with pepsin (Additional file 1: Table S1). To confirm if pepsin is involved IL-8 and IL-6 secretion in laryngeal carcinoma cells, pepsin was inhibited by pepstatin, an inhibitor of aspartate (acid) proteases, including pepsin, cathepsin D, and chymosin. IL-8 level decreased significantly by 31.4% and 34.92% (*P* = 0.027 and *P* = 0.007) and that of IL-6 decreased by 8.71% and 25.7% (*P* = 0.072 and *P* = 0.212) when pepsin was inhibited by pepstatin in Hep-2 and Tu212 cells, respectively (Fig. 2b). IL-8 may be more sensitive to pepsin than IL-6. Levels of IL-10, IL-1β, IL-12p70, and TNF were not significantly affected by pepstatin (Additional file 1: Table S2).

Furthermore, IL-8 expression was analyzed using laryngeal carcinoma specimens and immunohistochemistry. A significantly positive correlation was observed between pepsin treatment and the expression of IL-8 in laryngeal carcinoma tissue (*r* = 0.245, *P *= 0.022; Fig. 2c).

### Pepsin affected laryngeal squamous cell carcinoma cell morphology and EMT induction

**Fig. 3:**
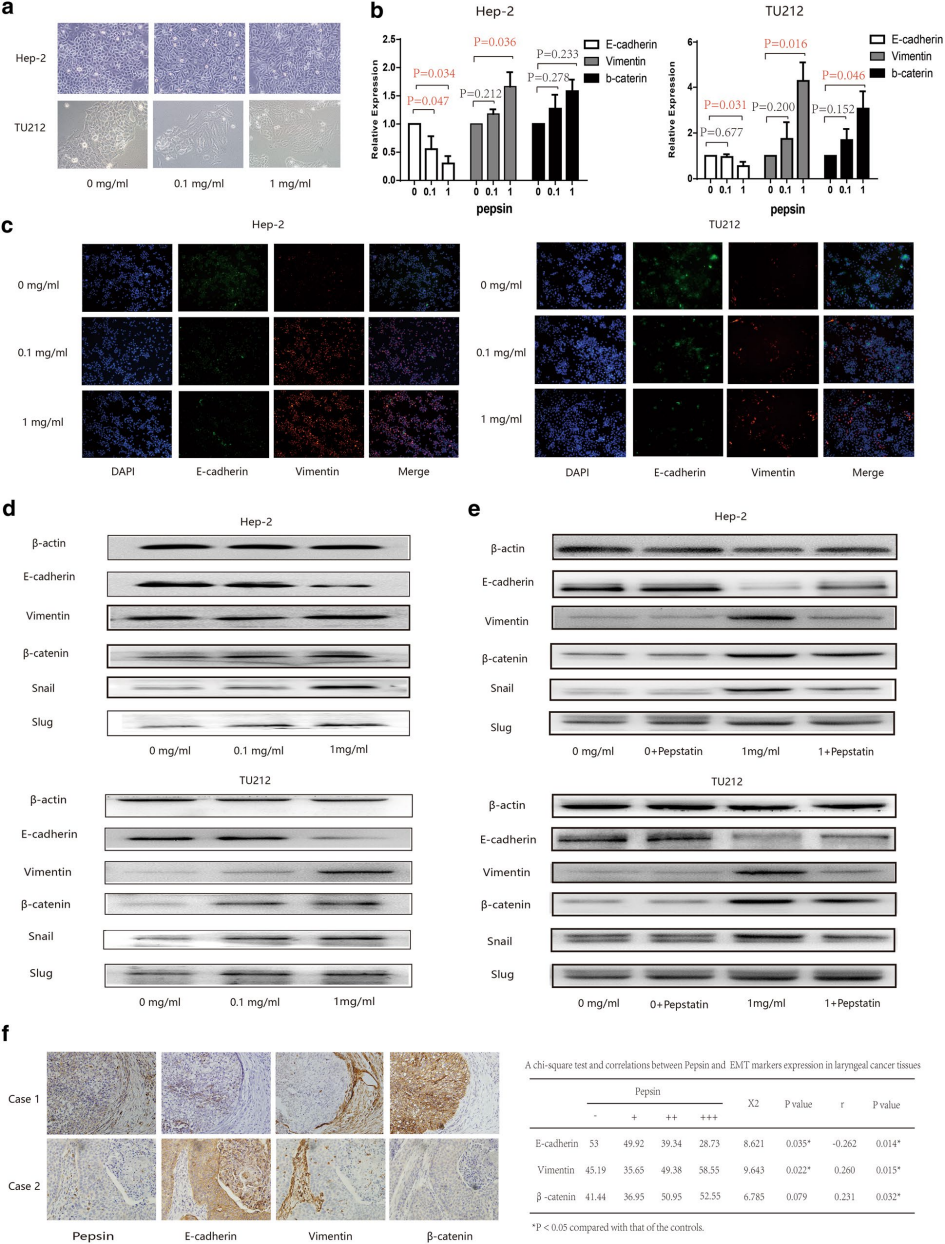
Expression of epithelial and mesenchymal markers in Hep-2 and Tu212 cells treated with pepsin. **a** Morphology of Hep-2 and Tu212 cells exposed to different pepsin concentrations is shown using phase contrast microscopy. **b** Expression levels of E-cadherin, vimentin, and β-catenin in Hep-2 and Tu212 cells exposed to different concentrations of pepsin analyzed using quantitative real-time PCR. **c** Effect of different pepsin concentrations on the expression of E-cadherin and vimentin in Hep-2 and Tu212 cells immunostained and analyzed using confocal microscopy (magnification, ×200). **d** Expression of E-cadherin, vimentin, β-catenin, snail, and slug in Hep-2 and Tu212 cells exposed to different concentrations of pepsin analyzed using western blotting. **e** Expression of E-cadherin, vimentin, β-catenin, snail, and slug in Hep-2 and Tu212 cells exposed to pepsin with/without pepstatin analyzed using western blotting. **f** Representative photomicrographs illustrating the immunohistochemical analyses for pepsin, E-cadherin, vimentin, and β-catenin in tissue specimens from two patients with laryngeal carcinoma (magnification, ×400). *P < 0.05 compared to that of the controls

A dramatic morphological change was observed in Hep-2 and Tu212 cells following pepsin stimulation. The typical cobblestone-like appearance of the squamous epithelium was replaced with a spindle-like fibroblastic morphology. After stimulation with 0.1 mg/ml pepsin for 5 days, the cells demonstrated fusiform growth and an obvious spindle-shaped morphology following stimulation with 1 mg/ml pepsin (Fig. 3a).

This phenomenon suggested that Hep-2 and Tu212 cell lines exposed to pepsin may undergo EMT. Further investigation revealed that the levels of E-cadherin in Hep-2 and Tu212 cells exposed to 0.1 mg/ml and 1 mg/ml pepsin decreased by 55.50% and 30.20%, (*P* = 0.034), and 95.33% and 55.00% (*P* = 0.037), respectively, compared to that of cells not treated with pepsin. In contrast, for Hep-2 and Tu212 cells exposed to 0.1 mg/ml and 1 mg/ml pepsin, the mRNA levels of vimentin increased by 1.18- and 1.66-fold (*P* = 0.036), and by 1.74- and 4.28-fold (*P* = 0.016), respectively. The mRNA levels of β-catenin in these two cell lines increased by 1.27- and 1.58-fold (*P* = 0.233), and by 1.69- and 3.07-fold (*P* = 0.046), respectively (Fig. 3b). Western blotting and immunofluorescence analyses of Hep-2 and Tu212 cells indicated that E-cadherin expression decreased and vimentin expression increased following exposure to different concentrations of pepsin (Fig. 3c, d). Furthermore, western blot analysis showed that β-catenin, snail, and slug were upregulated with increasing concentrations of pepsin. Treatment of Hep-2 and Tu212 cells with pepsin and its inhibitor pepstatin markedly increased E-cadherin expression but reduced vimentin and β-catenin expression compared to that of cells only exposed to pepsin (Fig. 3e).

Among 87 patients with laryngeal carcinoma, immunohistochemical staining for pepsin in the tissues was strongly positive in 11 patients (12.64%), moderately positive in 26 patients (29.89%), weakly positive in 28 patients (32.18%), and negative in 22 patients (25.29%). Furthermore, as shown in Fig. 3f, pepsin expression was associated with vimentin expression (*r* = 0.260, *P *= 0.015) and β-catenin expression (*r* = 0.231, *P* = 0.032), but inversely associated with E-cadherin expression (*r* = − 0.262, *P *= 0.014).

### Role of the IL-8/IL-8R axis in EMT and the proliferative and migratory capacities of laryngeal squamous cell carcinoma cell exposed to pepsin

**Fig. 4:**
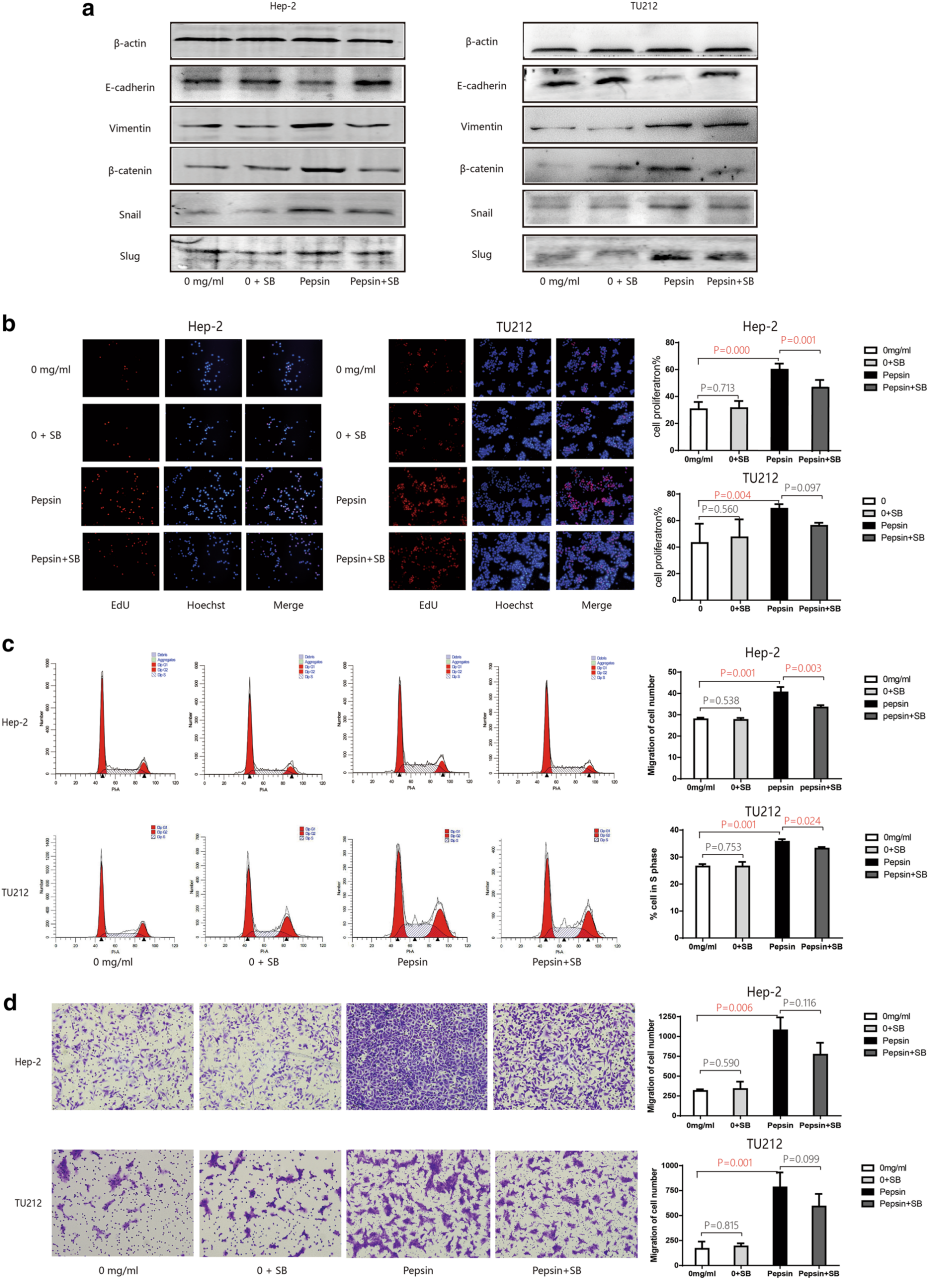
In vitro Hep-2 and Tu212 cells exposed to pepsin after the blockage of IL8 receptors. **a** Western blot analysis of E-cadherin, vimentin, β-catenin, snail, and slug expression in Hep-2 and Tu212 cells exposed to pepsin with or without SB225002 treatment to inhibit the IL-8 receptor CXCR2. **b** Effect of pepsin on Hep-2 and Tu212 cell proliferation with or without SB225002 treatment to inhibit the IL-8 receptor CXCR2. Cell proliferation was measured using Edu assays (magnification, ×100). **c** Representative histograms of cell cycle assays showing the percentage of pepsin-treated Hep-2 and Tu212 cells in S phase with or without SB225002 treatment to inhibit the IL-8 receptor CXCR2. **d** Migratory properties of Hep-2 and Tu212 cells exposed to pepsin with or without inhibition of the IL-8 receptor CXCR2 by SB225002 treatment analyzed using transwell migration assays, with the number of migrated Hep-2 and Tu212 cells shown (magnification, ×200). *P < 0.05 compared to that of the controls

To provide insight into the mechanism via which cytokine signals regulate pepsin-induced EMT in laryngeal carcinoma cells, SB225002 was added to the culture medium to block the IL-8 receptor CXCR2 [30]. Treatment of the Hep-2 and Tu212 cells exposed to pepsin with SB225002 markedly increased E-cadherin expression but reduced vimentin and β-catenin expression compared to that of cells not treated with the IL-8 inhibitor. Blocking of the IL-8 receptors with SB225002 did not affect slug expression, but markedly reduced snail expression (Fig. 4a).

Next, we assessed the effect of IL-8 on the proliferation capacity of Hep-2 and Tu212 cells using EdU assays and cell cycle experiments. Blocking of the IL-8 receptors in pepsin-treated Hep-2 and Tu212 cells significantly inhibited the proliferation capacity of the cells by 66.14% and 82.35% (*P* = 0.001 and *P* = 0.017), and reduced the percentage of cells in S-phase by 86.64% and 92.94% (*P* = 0.025 and *P* = 0.000) compared to that of cells not treated with the CXCR2 inhibitor (Fig. 4b, c). Transwell migration assays revealed that blockage of the IL-8 receptor CXCR2 reduced the migratory capacities of pepsin-treated Hep-2 and Tu212 cells by 72.06% and 75.38% (*P* = 0.018 and *P* = 0.002) compared to that of cells not treated with the CXCR2 inhibitor (Fig. 4d).

## Discussion

Pepsin can inhibit protective laryngeal proteins and contribute to laryngeal damage [31, 32]. Furthermore, Johnston et al. [33, 34] showed that pepsin can enter cells via receptors and may be stored in vesicles or transported within the cells to other complex organelles in a non-acid environment. Pepsin is reactivated once the pH of the environment is optimal and cause cell damage. As a result, the laryngeal mucosa may develop chronic inflammation and release inflammatory factors that foster the genetic evolution of preliminary neoplasia into mature cancers [35, 36]. Samuels [37] demonstrated the presence of pepsin in Barrett’s esophageal mucosa and the capacity of nonacid pepsin to alter the in vitro expression of inflammation and carcinogenesis markers in esophageal cells. Johnston et al. [21] also proposed that the promotion of cell proliferation and migration by pepsin may be associated with altered expression of tumor-related genes and microRNAs. Allen [38] used pepsin and 9,10-dimethyl-1,2-benzanthracene (DMBA) to stimulate the tumors of hamster cheek pouch and observed that tumor volume was significantly higher in the presence of pepsin than without pepsin; hence pepsin might be a potential cofactor required for tumor growth. Our recent study [25] indicated that patients with laryngeal carcinoma have the highest expression levels of pepsin, followed by patients with vocal cord leukoplakia and control subjects. Thus, pepsin may be associated with laryngeal cancer and might contribute to the development of laryngopharyngeal carcinogenesis. Further studies are required to elucidate the precise role of pepsin and the mechanism involved.

As the mean pH of the laryngopharynx is 6.8 [39, 40], the Hep-2 and Tu212 cells in the current study were exposed to pepsin at pH 7 and the proliferation and migration of the cells were assessed. Results from the current study provided strong evidence that pepsin promoted the proliferation and migration of Hep-2 and Tu212 cells in vitro. This suggests that pepsin functions as a tumor-promoting factor in laryngeal carcinoma. This is consistent with the results from correlation studies [22, 41, 42].

The Hep-2 and Tu212 cells transformed from typical polygons to fibroblast-like long spindles under pepsin stimulation, which was reminiscent of EMT. Thus, we hypothesized that pepsin stimulation might induce EMT of tumor cells and further promote tumor metastasis. Although Lorenz and colleagues [23] did not study laryngeal carcinoma, they proposed a correlation between EMT and reflux for the first time. In that study, the severity of reflux correlated with EMT scores, while the EMT grades of patients with expanded fistula were significantly higher than those without fistula expansion. Furthermore, Shellman et al. [24] showed that bile acids contribute to pharyngeal carcinogenesis via EMT. Several other studies have shown that repeated gastric acid and pepsin exposure stimulates the laryngeal epithelium mucosa to change the E-cadherin/β-catenin complex, which may be a potential risk factor for the development of laryngeal neoplasms [43–47].

To test our hypothesis that reflux of pepsin into the laryngopharynx can induce EMT in laryngeal carcinoma, we evaluated the expression of EMT markers in pepsin-stimulated Hep-2 and Tu212 cells. Our results showed that pepsin exposure reduced the expression of the epithelial marker E-cadherin and increased the expression of the mesenchymal markers vimentin and β-catenin, which was similar to the results observed with immunohistochemistry of laryngeal cancer tissue. Meanwhile, expression of the EMT transcription factors snail and slug correlated with pepsin concentration. Thus, we speculated that pepsin may change the expression of snail and slug via signaling pathways, inhibit E-cadherin expression, hinder adhesion of epithelial cells, and weaken the ability of cells to maintain their shapes [48]. Defect in E-cadherin expression may result in the release of β-catenin from the cell membrane and its accumulation in the cytoplasm, thereby connecting laryngeal mucosa epithelial cell damage with increase in epithelial permeability [49, 50]. The increase in vimentin protein expression improved cellular flexibility, mobility, and anti-immune capability [51], which induced EMT in laryngeal carcinoma.

Previous studies have proposed that inflammatory cytokines, such as transforming growth factor and interleukin, play crucial roles in EMT induction [52–54]. We analyzed cytokine concentrations in serum exposed to pepsin using CBA assays and observed a significant increase in IL-8 levels, which was dependent on the dose of pepsin. In addition, pepsin and IL-8 expression correlated positively in laryngeal carcinoma tissue. These observations are consistent with the results of Samuels et al. [26]. Furthermore, pepstatin is well known to be an inhibitor of aspartic proteinases such as pepsin, cathepsins D and E [55]. Kim observed that pepsin from extraesophageal reflux aggravates tonsil hypertrophy and pepstatin exerts a protective effect by inhibiting pepsin activity [56]. Our results showed that IL-8 level was reduced when pepsin was inhibited by pepstatin; this coincided with significant upregulation of epithelial markers and downregulation of mesenchymal markers.

**Fig. 5:**
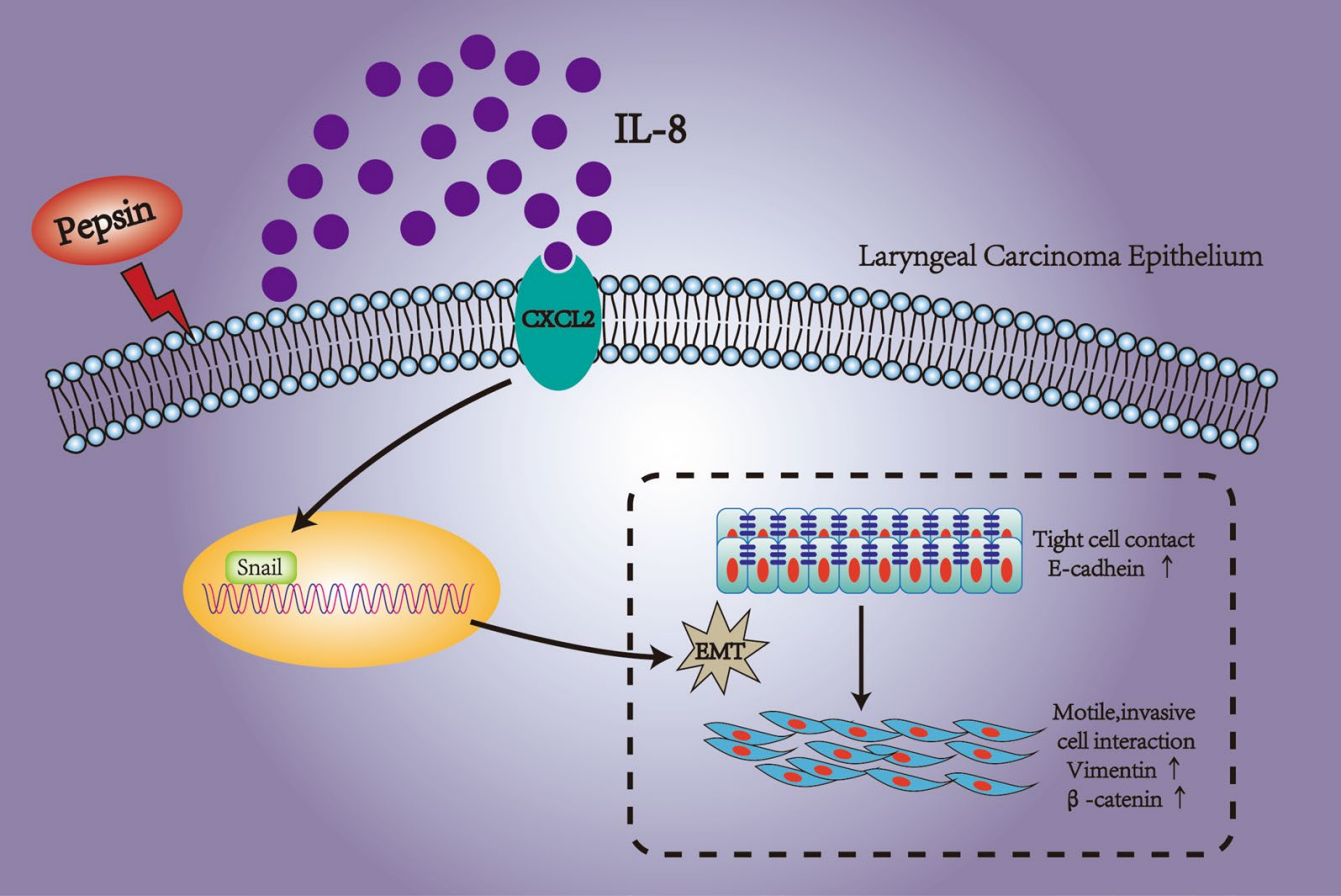
Pepsin may induce EMT in laryngeal carcinoma, underlining its potential role in enhancing laryngeal carcinoma proliferation and metastasis associated with IL-8 secretion

IL-8 is a multifunctional cytokine that participates in acute inflammation and as an extracellular signaling factor in the tumor microenvironment. Fernando et al. [57] demonstrated that IL-8 secreted by the human head and neck squamous cell carcinoma (HNSCC) cells undergoing EMT may play a crucial role in promoting tumor progression of HNSCC. To clarify whether IL-8 had a causal relationship with pepsin and EMT in Hep-2 and Tu212 cells, we suppressed CXCR2 using SB225002 [30] to inhibit the binding of IL-8 to CXCR2 during pepsin treatment. Cancer cells express the CXC receptors CXCR 1 and 2, and IL-8, the ligand for these receptors, stimulates migration and proliferation of these tumor cells [58]. We suppressed CXCR2 during pepsin treatment and observed that the pepsin-mediated promotion of cell proliferation and migration was inhibited. Blocking of CXCR2 expression during pepsin treatment partially restored the downregulation of epithelial markers and the upregulation of mesenchymal markers and transcription factors of EMT in Hep-2 and Tu212 cells. However, suppressed CXCR2 did not completely inhibit phenotype caused by pepsin, indicating either insufficient inhibition of IL8 or other inflammatory factors to play a role beside IL-8. Reflux causes epithelial cells to secrete chemokines, including IL-8, which leads to mucosal damage through inflammatory cell recruitment [59]. The inflammatory cells and cytokines found in tumors are more likely to contribute to tumor growth and progression, and deletion or inhibition of inflammatory cytokines inhibits the development of experimental cancer [36, 58]. Additionally, inflammatory cells can release chemicals, notably reactive oxygen species, that are actively mutagenic for nearby cancer cells, accelerating their genetic evolution toward states of heightened malignancy [35]. Studies demonstrated that pepsin induced the secretion of IL-8, which promoted EMT of laryngeal cancer (Fig. 5). EMT may reduce the protective ability of the cell epithelium by changing E-cadherin/β-catenin [48, 49], thereby destroying the histological barrier of the region invaded by tumor cells, facilitating cell separation and shedding, and finally contributing to cell migration and metastasis [60]. Inhibition of IL-8 binding to CXCR2 partially restored the effect of pepsin on laryngeal cancer cells.

## Conclusion

We propose that pepsin induced EMT in laryngeal carcinoma and provided new evidence for the role of LPR in laryngeal cancer tumorigenesis. LPR-associated pepsin may stimulate tumor cells to secrete IL-8 and activate the transcription factor snail to promote EMT in laryngeal cancer. Pepsin-induced EMT of laryngeal carcinoma provides a theoretical basis for understanding the pathogenesis of LPR in laryngeal carcinoma progression.

## Additional file

**Additional file 1: Table S1.** Change in inflammatory cytokine expression of Hep-2 and Tu212 cells exposed to different pepsin concentrations measured using CBA assays. **Table S2.** Change in inflammatory cytokine expression of Hep-2 and Tu212 cells exposed to pepsin with or without pepstatin measured using CBA assays.

## Abbreviations

LPR: laryngopharyngeal reflux
EMT: epithelial–mesenchymal transition
IL-8: interleukin-8
Hep-2: human laryngeal carcinoma cell line
Tu212: human laryngeal carcinoma cell line
