# Supplementary material for: Pepsin promotes IL-8 signaling-induced epithelial–mesenchymal transition in laryngeal carcinoma
Source: Cancer Cell Int. 2019 Mar 20;19:64. doi: 10.1186/s12935-019-0772-7 (PMC6425698; doi:10.1186/s12935-019-0772-7)
Supplement: Supplementary file 1 — Additional file 1: Table S1. Change in inflammatory cytokine expression of Hep-2 and Tu212 cells exposed to different pepsin concentrations measured using CBA assays. Table S2. Change in inflammatory cytokine expression of Hep-2 and Tu212 cells exposed to pepsin with or without pepstatin measured using CBA assays. [file 12935_2019_772_MOESM1_ESM.doc]

Table S1. Change in inflammatory cytokine expression of Hep-2 and Tu212 cells exposed to different pepsin concentrations measured using CBA assays

| Cell | IL | pepsin | | | F | *P* |
| --- | --- | --- | --- | --- | --- | --- |
|  |  | 0mg/L | 0.1mg/L | 1mg/L |  |  |
| TU212 | IL-8 | 24.97±5.23pg/ml | 35.86±13.76pg/ml | 66.75±20.75pg/ml | 6.527 | 0.021 |
|  | IL-6 | 39.14±17.22pg/ml | 61.80±37.13pg/ml | 93.54±78.20pg/ml | 0.863 | 0.469 |
|  | IL-10 | 1.09±0.56pg/ml | 1.14±0.31pg/ml | 0.97±0.55pg/ml | 0.095 | 0.911 |
|  | IL-1β | 1.1±0.65pg/ml | 1.1±0.69pg/ml | 1.18±0.39pg/ml | 0.02 | 0.98 |
|  | IL-12p70 | 1.06±0.55pg/ml | 1.27±0.35pg/ml | 1.38±0.44pg/ml | 0.392 | 0.692 |
|  | TNF | 0.93±0.35pg/ml | 0.93±0.3pg/ml | 1.47±0.92pg/ml | 0.825 | 0.482 |
| Hep-2 | IL-8 | 18.31±0.79pg/ml | 32.53±9.03pg/ml | 53.41±15.65pg/ml | 8.582 | 0.017 |
|  | IL-6 | 91.24±60.53pg/ml | 112.81±72.71pg/ml | 180.36±97.18pg/ml | 1.058 | 0.404 |
|  | IL-10 | 1.02±0.45pg/ml | 1.08±0.47pg/ml | 1.02±0.45pg/ml | 0.019 | 0.981 |
|  | IL-1β | 0.6±0.23pg/ml | 0.8±0.12pg/ml | 1.03±0.84pg/ml | 0.562 | 0.597 |
|  | IL-12p70 | 0.59±0.28pg/ml | 0.75±0.19pg/ml | 0.69±0.03pg/ml | 0.52 | 0.619 |
|  | TNF | 0.97±0.91pg/ml | 0.88±0.61pg/ml | 0.60±0.17pg/ml | 0.264 | 0.776 |

Table S2. Change in inflammatory cytokine expression of Hep-2 and Tu212 cells exposed to pepsin with or without pepstatin measured using CBA assays

| Cell | IL | pepsin | | |  | F | *P* |
| --- | --- | --- | --- | --- | --- | --- | --- |
|  |  | 0mg/L | 0.1mg/L | 1mg/L | 1mg/L+pepstatin |  |  |
| TU212 | IL-8 | 35.30±10.99pg/ml | 32.34±9.9pg/ml | 95.27±14.59pg/ml | 62±27.22pg/ml | 8.754 | 0.007 |
|  | IL-6 | 168.54±30.55pg/ml | 151.46±59.86pg/ml | 251.72±45.68pg/ml | 187.03±75.47pg/ml | 1.874 | 0.212 |
|  | IL-10 | 2.54±0.88pg/ml | 2.78±0.77pg/ml | 2.39±0.26pg/ml | 3.08±0.17pg/ml | 0.729 | 0.563 |
|  | IL-1β | 5.29±2.06pg/ml | 6.62±2.31pg/ml | 6.1±2.54pg/ml | 8.37±1.22pg/ml | 0.772 | 0.542 |
|  | IL-12p70 | 3.34±0.8pg/ml | 3.07±0.8pg/ml | 3.09±0.77pg/ml | 3.11±0.12pg/ml | 0.098 | 0.959 |
|  | TNF | 2.44±0.39pg/ml | 2.77±0.58pg/ml | 3.21±0.62pg/ml | 2.76±0.68pg/ml | 0.890 | 0.487 |
| Hep-2 | IL-8 | 22.21±4.49pg/ml | 25.12±7.57pg/ml | 76.95±27.24pg/ml | 52.79±20.52pg/ml | 5.264 | 0.027 |
|  | IL-6 | 181.48±47.36pg/ml | 190.56±44.15pg/ml | 281.43±36.35pg/ml | 256.93±53.80pg/ml | 3.452 | 0.072 |
|  | IL-10 | 2.24±0.65pg/ml | 2.04±0.34pg/ml | 2.23±0.59pg/ml | 2.48±0.37pg/ml | 0.392 | 0.762 |
|  | IL-1β | 9.69±1.09pg/ml | 9.48±2.33pg/ml | 9.09±1.11pg/ml | 9.89±0.48pg/ml | 0.171 | 0.913 |
|  | IL-12p70 | 3.51±0.53pg/ml | 2.77±0.49pg/ml | 2.71±0.3pg/ml | 2.92±0.39pg/ml | 2.134 | 0.174 |
|  | TNF | 2.21±1.12pg/ml | 1.96±0.65pg/ml | 2.4±0.53pg/ml | 1.74±0.54pg/ml | 0.454 | 0.72 |
